# Integrative taxonomy confirms that *Gregarina garnhami* and *G. acridiorum* (Apicomplexa, Gregarinidae), parasites of *Schistocerca gregaria* and *Locusta migratoria* (Insecta, Orthoptera), are distinct species

**DOI:** 10.1051/parasite/2021009

**Published:** 2021-02-23

**Authors:** Isabelle Florent, Marie Pierre Chapuis, Amandine Labat, Julie Boisard, Nicolas Leménager, Bruno Michel, Isabelle Desportes-Livage

**Affiliations:** 1 Molécules de Communication et Adaptation des Microorganismes (MCAM, UMR 7245 CNRS), Département Adaptations du vivant (AVIV), Muséum National d’Histoire Naturelle, CNRS CP 52 57 rue Cuvier 75231 Paris Cedex 05 France; 2 CBGP, Univ Montpellier, CIRAD, INRAE, Institut Agro, IRD 34060 Montpellier France; 3 CIRAD, UMR CBGP 34398 Montpellier France; 4 Structure et instabilité des génomes (STRING UMR 7196 CNRS/INSERM U1154), Département Adaptations du vivant (AVIV), Muséum National d’Histoire Naturelle, CNRS, INSERM CP 26 57 rue Cuvier 75231 Paris Cedex 05 France

**Keywords:** Gregarines, Orthoptera, Species delimitation, SSU rDNA phylogeny, Phenotypic plasticity, Biodiversity

## Abstract

Orthoptera are infected by about 60 species of gregarines assigned to the genus *Gregarina* Dufour, 1828. Among these species, *Gregarina garnhami* Canning, 1956 from *Schistocerca gregaria* (Forsskål, 1775) was considered by Lipa et al. in 1996 to be synonymous with *Gregarina acridiorum* (Léger 1893), a parasite of several orthopteran species including *Locusta migratoria* (Linné, 1758). Here, a morphological study and molecular analyses of the SSU rDNA marker demonstrate that specimens of *S. gregaria* and specimens of *L. migratoria* are infected by two distinct *Gregarina* species, *G. garnhami* and *G. acridiorum,* respectively. Validation of the species confirms that molecular analyses provide useful taxonomical information. Phenotypic plasticity was clearly observed in the case of *G. garnhami*: the morphology of its trophozoites, gamonts and syzygies varied according to the geographical location of *S. gregaria* and the subspecies infected.

## Abstract (fr)

Les orthoptères sont parasités par environ soixante espèces de grégarines affiliées au genre *Gregarina* Dufour, 1828. Parmi ces espèces *Gregarina garnhami* Canning, 1956 décrite chez *Schistocerca gregaria* (Forskål, 1775), a été mise en synonymie par Lipa et al. en 1996 avec *Gregarina acridiorum* (Léger 1893), parasite de plusieurs espèces d’orthoptères dont *Locusta migratoria* (Linné, 1758). Ici, une étude morphologique et des analyses moléculaires du marqueur SSU rDNA démontrent que les spécimens de *S. gregaria* et ceux de *L. migratoria* sont infectés par 2 espèces distinctes de grégarines, *Gregarina garnhami* et *Gregarina acridiorum*, respectivement. La validation de ces espèces confirme l’importance des informations fournies par les analyses moléculaires dans les études taxonomiques. Une plasticité phénotypique a été clairement observée dans le cas de *G. garnhami :* la morphologie de ses trophozoïtes, gamontes et syzygies varie selon la localisation géographique et la sous-espèce de *S. gregaria* infectée.

## Introduction

Gregarines are a heterogeneous group of apicomplexan parasites that infect a very wide range of non-vertebrate hosts, in which they mostly occupy intestinal tracts and coelomic spaces [17]. The biodiversity of gregarines currently corresponds to 1600-1700 formally described species [32], but according to experts in the field, this number may be vastly underestimated [1, 17]. Recent metagenomic surveys of terrestrial soils and marine environments further confirmed the high occurrence and abundance of gregarine-like sequences in these environments that remain to be ascribed to formally described species [15, 16, 28]. In the past, ascribing gregarine species assignations was based on combinations of morphological and behavioral features including parasitic life traits (host and host range specificities), the different locations occupied by the parasite in hosts (i.e. intestine or coelom), descriptions of life-cycle development stages (morphological measurements, duration of the stages, scanning and transmission electron microscopy), gamont pairing (frontal, lateral, caudo-frontal), and modes of gametocyst dehiscence [11, 17, 26]. The increasing use of molecular data in recent decades has led to the confirmation, but also sometimes to the revision of the taxonomic and phylogenetic view we have of gregarines, and has revealed that some species that were once considered distinct are in fact the same [19] or, the reverse, novel cryptic species, i.e. morphologically indistinguishable but clearly distinct at the molecular level [30].

Orthoptera (Ensifera (crickets and katydids) and Caelifera (grasshoppers, ground-hoppers and pygmy mole crickets)) are reported to be parasitized by about 60 species assigned to the genus *Gregarina* Dufour, 1828 (see [17] for a recent, extensive review of the literature). Based on morphological descriptions, some gregarine species have been found to be restricted to one host family or superfamily, while others seem to have the capacity to infect a wide range of hosts distributed worldwide [14, 17, 36, 37]. Problems of identification based on morphological characters likely arose from phenotypic plasticity in response to wide-range host species and/or other contrasted environmental conditions. As a result, species delimitation within the genus *Gregarina* has been the subject of debate, with confusion, descriptions and synonymies, in particular for gregarines that infect the Caelifera suborder, as illustrated below. Species delimitation is, however, a global and recurrent issue in protistology [6].

In 1893, Léger described *Clepsidrina acridiorum* [24], which, a few years later, was termed *Gregarina* by Labbé (1899) [21]. This parasite was found in Acridoidea collected in Algeria [24]. As the infected specimens belonged to different genera of Caelifera (*Truxalis*, *Pamphagus*, *Sphingonotus*), Léger concluded that “other acridians from Africa should be investigated for potential *G. acridiorum* infections” [24]. Interestingly, he noticed that *G. acridiorum* was not found in the desert locust, *Schistocerca gregaria* [24]. Later in 1956, Canning described a gregarine she named *Gregarina garnhami*, sampled from this *S. gregaria* host [7]. Interestingly, *G. garnhami* was also reported by the same author in both the migratory locust, *L. migratoria* and in the Egyptian locust, *Anacridium aegyptium* [7]. According to data in the literature, *G. acridiorum* and *G. garnhami* share common morphological and behavioral characteristics, such as their development in the midgut of their hosts, a small globular epimerite, stout bodied gamonts, and barrel-shaped (or dolioform) oocysts [7, 23, 27]. In 1996, Lipa et al. concluded that the species described in 1956 by Canning in *S. gregaria* was in fact *G. acridiorum* [27]. This interpretation was supported by the fact that in 1956, Canning had not been aware of the existence of *G. acridiorum* [27]. *Gregarina acridiorum* has been reported in a range of Orthoptera hosts (Ensifera and Caelifera: Acrididae, Tetrigidae) including *L. migratoria* and *A. aegyptium* [12, 27], two species also described as hosts of *G. garnhami* [7]. Consequently, the two acridian species could be infected by the two gregarines species.

*Gregarina acridiorum* and *G. garnhami* also closely resemble *Gregarina rigida* (Hall, 1907) Ellis, 1913, described in a broad range of widely distributed orthopteran hosts [17] and also similar to *Gregarina ronderosi*, a parasite of the argentine grasshopper *Dichroplus elongatus* [22]. The developmental and morphological similarities of these four gregarines question their species definition as well as their host specificities and indeed, based on these similarities, in 1968, Corbel even proposed that *G. rigida* and *G. acridiorum* were the same [13]. Table 1 lists the main biological and morphological characters of these four very similar gregarines of acridians, plus data concerning a fifth species, *Gregarina caledia* (nomen nudum), a parasite of the Australian grasshopper *Caledia captiva*, described in the PhD Thesis of Jennifer Ann Ninham (1995) and reported to be very similar to *G. garnhami* [30]. Table 1 illustrates how tenuous some of these differences can be when these five gregarines of acridians are compared. The limited availability of DNA sequences corresponding to these species is an obstacle to the resolution of these controversies (only partial SSU rDNA sequences (1210 bp) are available for *G. caledia* (L31799) and *Gregarina chortiocetes* (L31841)). The latter species, an intestinal parasite of *Chortiocetes terminifera,* is however poorly described at the morphological level [30].

**Table 1:** Morphological differences between five very similar gregarines of acridians reported in the literature. This table is based on individual descriptions provided by the authors of [7, 21, 22, 24, 30]; see also [17]. *D*, diameter; *L*, length; *W*, width; TL, total length.

Gregarine	*Gregarina acridiorum* (Léger, 1893) Labbé, 1899 [24] [21]	*Gregarina garnhami* Canning, 1956 [7]	*Gregarina rigida* (Hall, 1907) Ellis, 1913 [17]	*Gregarina ronderosi,* Lange & Wittenstein, 2002 [22]	*Gregarina caledia,* Ninham, 1995 [30]
Hosts	Caelifera: Acrididae, Tetrigidae; Ensifera: Tettigoniidae	Caelifera: Acrididae	Caelifera: Acrididae; Ensifera: Tettigoniidae	Caelifera: Acrididae	Caelifera: Acrididae
Infected sites in hosts	Midgut	Early stages in gastric ceca and occasionally in the midgut; gamonts in the midgut	Early stages in gastric ceca, near the anterior end of the midgut	Trophozoites, solitary or associated gamonts in gastric ceca and gut; gametocysts in the hindgut	Trophozoites, solitary or associated gamonts in gastric ceca and midgut; gametocysts in the hindgut
**Trophozoites – gamonts**					
Gamonts	Gamonts: cylindrical, ovoid in older forms, endocyte yellow orange. *L*: ~ 400 μm, *W*: 160 μm	Gamonts: rather stout bodied in older forms, endocytes are pale yellow. *L*: 250–554 μm	Gamonts: rather stout bodied, endocytes are brownish orange. *L*: 250–750 μm *W*: 130–210 μm	Trophozoite (epimerite): *L*: 10.4–275 μm, more slender than gamonts; Gamonts: rather stout bodied, endocytes are pale yellow. *L*: 80–348 μm	Gamonts: pale-yellow, ovoid then cylindrical *L*: 180–264 μm *W*: 60–70 μm Mean: 222 μm × 65 μm
Association Length	TL: up to 1000 μm	TL: 500–1110 μm	TL: up to 1425 μm (average: 550 μm). Protomerite smaller in the satellite than in the primite	TL: 160–700 μm (average: 425 μm). Primites and satellites are similar in size and shape	TL: 515 μm. Primites and satellites are similar in size. Also seen: primite with 2 small satellites
Epimerite	Small, spherical with a short stalk.	Small, globular with a short stalk.	Small spherical hyaline knob.	Conical when attached, globular and smaller in free trophozoites	Globular
Protomerite	Sub-globular in primites, depressed at the anterior end in satellites.	Conical in young stages, subspherical in older stages, widest at the septum, tapering towards the anterior end, flattened in satellite.	Somewhat flattened, 3 times wider than long, generally less constriction at septum more or less indistinct.	Sub-globular in primites, depressed at anterior end in satellites, less flattened than in *G. garnhami*	Rounded anterior end
Deutomerite	Cylindrical, rounded posterior end	Cylindrical or rounded, with sharply pointed posterior end	Cylindrical or barrel-shaped, little wider than protomerite, broadly rounded end or flattened “cornered” extremity	Cylindrical, rounded posterior end, wider than protomerite, barrel-shaped in older forms	Cylindrical, in small gamonts, wider than in protomorites and rounded in older forms
**Gametocysts – oocysts**					
Gametocysts	*D*: 500 μm, thick ectocyst	*D*: 114–470 μm (exclusive of the ectocyst)	*D*: 300 μm in average. Yellow orange color. Thick ectocyst	*D*: 96–376 μm. Thick ectocyst	*D*: 228–312 μm (mean 270 μm). Yellow orange color. Ectocyst (24–100 μm thick)
Basal discs	Yellow orange	Yellow orange	Not mentioned	Orange	Orange
Sporoducts	12–15, with a swollen basal part, *L* > 1/2 cyst diameter	8, *L*: 1/3 cyst diameter (without ectocyst)	10 or more, short	12–15, *L*: up to 60 μm	5 to more than 10
Oocysts (sporocysts)	Dolioform*, double wall	Dolioform*, thick wall	Barrel-shaped*	Dolioform* or Barrel-shaped*	Barrel-shaped*
	7.6 μm × 3.3 μm	6.5–7 μm × 4 μm	8 μm × 5 μm	5 μm × 3.2 μm	12 μm × 6 μm

* Depending on the authors, the terms “dolioform” and/or “barrel-shaped” were used to describe the shape of oocysts. Note also that oocysts were called sporocysts in all these historical descriptions.

In 2002, Lange & Wittenstein indicated that: “given the great similarity of *Gregarina* spp. associated with Acrididae, it would probably be very informative to study, at the molecular level, as many species as possible” [22]. To achieve this objective, we combined morphological and molecular data to better explore the species boundaries of gregarines that infect two orthopteran Acrididae hosts, *S. gregaria* (Forsskål, 1775) and *L. migratoria* (Linné, 1758). These two hosts are locusts, i.e. grasshoppers that can form dense migrating swarms, that are often destructive to agriculture, through an extreme form of density-dependent phenotypic plasticity, known as phase polyphenism [3, 41]. Here we sought to determine whether they are infected by the same or distinct gregarine species, as the information in the current literature is not congruent [7, 12–14, 21, 24, 27].

Morphological observations of the developmental stages of gregarines from *L. migratoria* and two subspecies of *S. gregaria* were performed and completed with the sequencing of their SSU rDNA loci. The results revealed clear molecular differences in this genetic marker, despite extremely similar morphological features, strongly supporting the hypothesis that these two acridian hosts are not infected by the same gregarine species. Some subtle morphological differences have also been identified between the two gregarine species.

## Materials and methods

### Collection of hosts and isolation of parasites

Specimens of *L. migratoria* (Linné, 1758) were obtained from the vivarium belonging to the French National Museum of Natural History (French acronym MNHN) (Source uncertain; time of establishment > 15 years, regularly replenished from Insect Raising SARL (2, Chemin Champthiaud, 25410 Dannemarie-sur-Crète, France). Two sub-species of the desert locust, *S. gregaria,* were studied: *S. gregaria gregaria* (Forsskål, 1775) and *S. gregaria flaviventris* (Burmeister, 1838), isolated in distinct regions along a north–south axis in Africa [10, 41]. The *S. g. gregaria* insects came from either a long-standing laboratory strain belonging to the team involved in molecular developmental physiology and signal transduction of the Department of Biology of Leuven University, Belgium (https://bio.kuleuven.be/df/jv; geographical source: Mauritania; time since establishment: several decades) or a long-standing laboratory strain acquired from the National Anti-Locust Centre in Agadir, Morocco, regularly replenished with individuals sampled in the field (geographical source: between Draa wadi and the Dakhla region; time of establishment: from the 1990s to 2014). *Schistocerca gregaria gregaria* egg pods from the two strains were received at the SEPA platform in CBGP on May 30 and June 12, 2014, respectively, and hatchlings were crowd-reared before treatment (July 17 and 23, 2014) in a breeding chamber at 32 °C, with 50% humidity, with a 12 h:12 h photoperiod, and fed with seedling wheat, supplemented by wheat bran for adults. The *S. g. flaviventris* insects came from a natural population in Tankwa Karoo National Park, South Africa (20.03° E; −32.23°), in which 70 nymphs were collected on February 23, 2014 and taken to the SEPA platform in CBGP for two generations of maintenance before treatment on July 11 and July 18, 2014, in the same breeding chamber and under the same conditions.

The hosts used in this study and the dates of sampling for biological analyses are listed in Table 2. All acridian specimens were anesthetized with chloroform. Their digestive tract was dissected in 0.22 μm-filtered sterile PBS 1X and gamonts (solitary or in syzygies) and gametocysts were isolated from ceca and midguts (*S. gregaria*) or only midguts (*L. migratoria*) using tweezers and sterile elongated Pasteur pipettes, under a stereomicroscope. Gametocysts were also occasionally isolated from insect feces and kept at room temperature to observe dehiscence. All isolated gregarines were washed at least three times in 0.22 μm-filtered sterile PBS 1X to eliminate host tissue and environmental bacteria prior to being used for microscopic observations, fixed for scanning electron microscopy, or stored as cell pellets at −20 °C prior to genomic DNA extraction.

**Table 2:** Acrididae hosts used in this study, sampling dates, host status and sampled gregarines. “Sick” hosts died rapidly (within days) in laboratory conditions in contrast to “healthy” hosts that were maintained for weeks.

Acrididae host/designation in study	Source	Host status	Gregarines sampled
*Schistocerca gregaria gregaria* (2014)/**SG-M**	Long-standing laboratory strain from CNLA Agadir, Morocco	Sick	Young trophozoites in ceca, gamonts, syzygies and gametocysts in the midgut, occasionally gametocysts in feces; high infection level
*Schistocerca gregaria gregaria* (2014)/**SG-B**	Long-standing laboratory strain from KU Leuven, Belgium	Healthy	Young trophozoites in ceca, gamonts, syzygies and gametocysts in the midgut, occasionally gametocysts in feces; high infection level
*Schistocerca gregaria flaviventris* (2014)/**SG-SA**	Natural population from Tankwa Karoo National Park, South Africa	Sick	Young trophozoites in ceca, gamonts, syzygies and gametocysts in the midgut, occasionally gametocysts in feces; high infection level
*Locusta migratoria* (2012, 2014, 2015)/**LM-M**	Long-standing laboratory strain from MNHN Vivarium, France	Healthy	Gamonts, syzygies and gametocysts in the midgut, occasionally gametocysts in feces; mild infection level

### Morphological studies

Isolated parasites were first observed on slides using light microscopy. Images were acquired using a Nikon DXM 1200C camera and a micrometric slide to set the scales, and the images were processed using ImageJ software (https://imagej.nih.gov/ij/). In parallel, pools of isolated and washed gamonts and gametocysts and relevant sections of infected acridian ceca and midguts were prepared for scanning electron microscopy (SEM). After appropriate washing in 0.22 μm-filtered sterile PBS 1X, the samples were fixed in 5% (v/v) glutaraldehyde in 0.2M cacodylate buffer (pH 7.2) at 4 °C for 6–12 h then washed twice in 0.2M cacodylate buffer (pH 7.2) before undergoing successive series of dehydration in 50, 70, 90 and 100% ethanol. Samples were critical point-dried in liquid CO_2_ (Emitech K850, Quorum Technologies, Lewes, United Kingdom) then coated with 20 nm gold (JFC-1200 Fine coater, JEOL, Tokyo, Japan). Samples were then examined with a Hitachi Scanning Electron Microscope SU3500 Premium (Hitachi, Tokyo, Japan), as previously described [2]. Quantitative measurements were length and width at the different life stages, including length of protomerites and deutomerites for trophozoites and gamonts.

### Statistical tests

In order to compare the averages of the measurements carried out for the gregarines infecting either *S. gregaria* or *L. migratoria* hosts, statistical tests were performed as follows. For the group of measurements with *n* = 18, we used a Shapiro–Wilk Test to assess the normality of the data, which established normality. For this sample and all the other groups of measurements tested with *n* > 30, we used parametric tests. First, a Fisher test was conducted to test the homoscedasticity of the variances within the groups. When homoscedasticity was retrieved, we conducted a Student’s *t*-test to compare the means of each group. When homoscedasticity was not retrieved, we then used a Welch’s *t*-test. Analyses were performed using R software.

### DNA extraction and sequencing

Total genomic DNA was extracted from pools of parasites (gamonts or gametocysts), isolated from individual host specimens as indicated in Table 3, using standard phenol-chloroform extractions [34] or MasterPure^TM^ Complete DNA and RNA Purification kits (Epicentre Biotechnologies, Madison, WI, USA), as previously described [35]. Isolated nucleic acids were subsequently used as templates in standard PCR reactions designed to amplify most of the SSU rDNA loci (V1–V8) [18], using forward WL1 – 5′–GCGCTACCTGGTTGATCCTGCC–3′ and reverse EukP3 5′–GACGGGCGGTGTGTAC–3′ primers, as previously described [35]. After confirmation of the appropriate amplicon size by agarose-gel electrophoresis, PCR products were purified using an Illustra^TM^ GFX^TM^ PCR DNA and Gel Band Purification Kit (GE Healthcare, France), and cloned into a pGEM^®^-T Easy Vector (Promega, Madison WI, USA), as previously described [35, 39]. DNA sequences were obtained by Sanger technology (Beckman Coulter Genomics, Takeley, United Kingdom) from positive clones selected by PCR using the T7 and Sp6 universal primers that flank the pGEM^®^-T Easy Vector cloning site, as previously described [39]. In addition to using T7 and Sp6 as sequencing primers, several internal primers were used (LWA3 5′–AAACTTAAAGGAATTGACGG–3′; PIF4F 5′–CCGTTACTTTGAGCAAATTGG–3′; PIF4R 5′–CTTAGAATTTCACCTCTCTCC–3′). SSU rDNA loci were then aligned and assembled from raw data using MEGA X [20]. The 43 novel sequences were deposited in the European Nucleotide Archive (ENA) database under accession numbers: LR814064–LR814106 (http://www.ebi.ac.uk/ena/data/view/LR814064-LR814106).

**Table 3:** Gregarine specimens isolated for molecular investigation.

Host	Geographical origin and collection date	Number of isolated parasite stages	gDNA preparation (name, method)	Parasite clones (clone designations)
*Locusta migratoria*	MNHN 2012	Gamonts (50)	LW, Phenol chloroform	LM1.01.M.2012-1
*Locusta migratoria*	MNHN 2014	Gamonts (50)	JF, MasterPure	LM2.01.M.2014-2
*Locusta migratoria*	MNHN 2015	Gametocysts (20)	JS310, MasterPure	LM3.01.M.2015-3
				LM3.02.M.2015-4
				LM3.03.M.2015-5
				LM3.04.M.2015-6
				LM3.05.M.2015-7
*Locusta migratoria*	MNHN 2015	Gametocysts (17)	JS311, MasterPure	LM4.01.M.2015-8
				LM4.02.M.2015-9
				LM4.03.M.2015-10
				LM4.04.M.2015-11
*Locusta migratoria*	MNHN 2015	Gametocysts (13)	JS312, MasterPure	LM5.01.M.2015-12
				LM5.02.M.2015-13
				LM5.03.M.2015-14
				LM5.04.M.2015-15
*Locusta migratoria*	MNHN 2015	Gametocysts (13)	JS313, MasterPure	LM6.01.M.2015-16
				LM6.02. M.2015-17
				LM6.03. M.2015-18
				LM6.04. M.2015-19
				LM6.05. M.2015-20
*Locusta migratoria*	MNHN 2015	Gametocysts (17)	JS314, MasterPure	LM7.01. M.2015-21
				LM7.02. M.2015-22
				LM7.03. M.2015-23
*Schistocerca gregaria flaviventris*	South Africa 2014	Gamonts (10) and Gametocysts (10)	JS260, MasterPure	SG1.01.SA.2014-24
				SG1.02.SA.2014-25
				SG1.03.SA.2014-26
				SG1.04.SA.2014-27
*Schistocerca gregaria flaviventris*	South Africa 2014	Gametocysts (9)	JS261, MasterPure	SG2.01.SA.2014-28
				SG2.02.SA.2014-29
				SG2.03.SA.2014-30
				SG2.04.SA.2014-31
				SG2.05.SA.2014-32
*Schistocerca gregaria flaviventris*	South Africa 2014	Gamonts (~250)	JS269, MasterPure	SG3.01.SA.2014-33
				SG3.02.SA.2014-34
				SG3.03.SA.2014-35
*Schistocerca gregaria gregaria*	Belgium 2014	Gamonts (~200)	JS267, MasterPure	SG4.01.B.2014-36
				SG4.02.B.2014-37
				SG4.03.B.2014-38
				SG4.04.B.2014-39
				SG4.05.B.2014-40
				SG4.06.B.2014-41
				SG4.07.B.2014-42
*Schistocerca gregaria gregaria*	Morocco 2014	Young trophozoites in ceca (~400)	JS272, MasterPure	SG5.01.Ma.2014-43

### Phylogenetic analyses

Using maximum likelihood (ML) and Bayesian methods, phylogenetic trees were built from 69 sequences from gregarines infecting either *S. gregaria* (20 sequences), *L. migratoria* (23 sequences), a range of different insect hosts (22 sequences) or marine crustaceans, chosen as the gregarine outgroup specimen (4 sequences) [11, 30, 35]. Using a previously published alignment [35], the new gregarine sequences were added manually to yield a confident alignment of 1433 positions, after selection of conserved blocks defined using Gblocks 0.91b [8] (parameters used: Minimum Number Of Sequences For A Conserved Position: 35; Minimum Number Of Sequences For A Flanking Position: 58; Maximum Number Of Contiguous Nonconserved Positions: 8; Minimum Length Of A Block: 3; Allowed Gap Positions: With Half Use Similarity Matrices: Yes). A GTR substitution model with gamma-distributed rate variation across sites and a proportion of invariant sites was suggested as the best-fit model by MEGA X [20]. A Bayesian phylogenetic tree was constructed with MrBayes v3.2.3 [33] using lset *n* = 6 rates = invgamma parameters; Monte Carlo Markov Chain parameters were mcmc ngen = 100 00 000 relburnin = yes burninfrac = 0.25 samplefreq = 1000 printfreq = 10 000 nchains = 4 nruns = 2. A consensus tree was constructed from the post burn-in trees and posterior probabilities were calculated in MrBayes. Posterior probabilities > 0.95 were considered strong support. Maximum likelihood analyses were performed using RAxML version 8.2.12 [40] using the GTR + G + I model; bootstraps were estimated from 1000 replicates. Bootstrap percentages > 75% were considered good support. Trees were visualized and edited with FigTree and Inkscape.

### Estimates of genetic divergence between and within groups

The numbers of base differences per site from averaging over all sequence pairs between and within each group were calculated using MEGA X [20]. This analysis involved 44 nucleotide sequences: 20 from gregarines that infect *S. gregaria,* 23 from gregarines that infect *L. migratoria*, and the sequence of *G. caledia* that infects *C. captiva* (L31799). For each sequence pair, all ambiguous positions were removed (pairwise deletion option) leaving a total of 1784 positions in the final dataset. From this dataset, we also constructed a minimum spanning network to analyze the relationships among the cloned SSU rDNA sequences using POPART [25].

## Results

**Figure 1:**
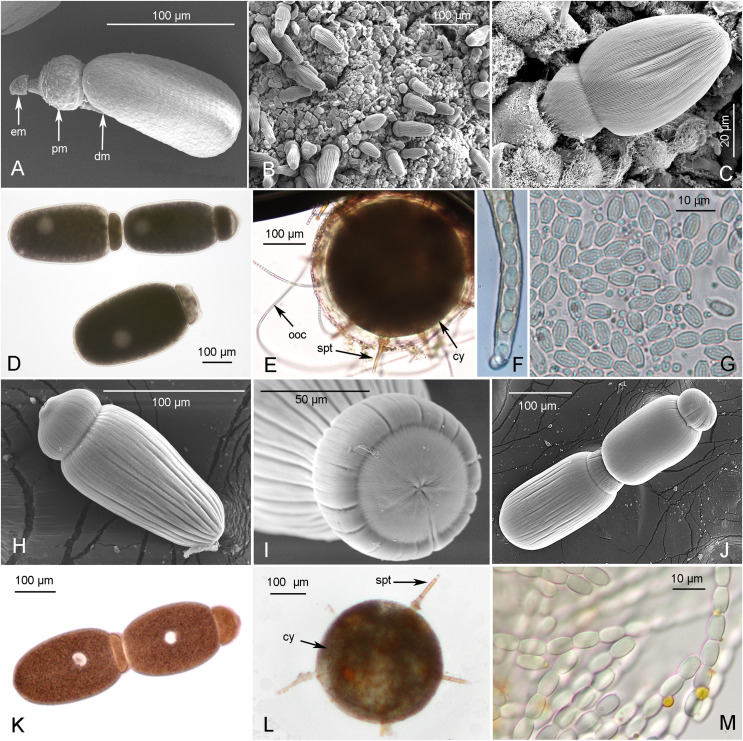
Scanning Electron Microscopy (A–C, H–J) and photonic imaging (D–G, K–M) of gregarines infecting *S. gregaria* (A–G) and *L. migratoria* (H–M). *S. gregaria* gregarines: A, young trophozoite (epimerite (em) protomerite (pm) and deutomerite (dm)), (South Africa); B, intestinal tract infected by numerous gregarines (Morocco); C, gregarine encased in an intestinal host cell, enlargement of B (Morocco); D. Solitary gamont and syzygy (Belgium); E. Gametocyst form (cy) with developed sporoducts (spt) releasing oocyst chains (ooc); F, zoom on sporoduct extremity showing enclosed oocysts; G. released oocysts. *L. migratoria* gregarines: H, solitary gamont detached from intestinal host cell; I. zoom on gamont protomerite; J–K, gamonts associated in syzygies; L, Gametocyst form (cy) with developed sporoducts (spt); M. released oocysts. Scales are given for each figure.

Gregarines isolated from the intestinal tracts of various acridian *S. gregaria* and *L. migratoria* host specimens (Table 2) were mostly located between the host intestine epithelial cells and digested food material. In addition, in all *S. gregaria* specimens, young trophozoite stages were invariably observed in the host’s ceca, whereas this was never observed in *L. migratoria*. Occasionally, gametocysts were also isolated from insect feces and kept at room temperature to observe dehiscence. The observed stages were trophozoites, solitary gamonts, gamonts associated in caudo-frontal syzygies, and gametocysts enclosing oocysts or emitting them as chains through sporoducts (Fig. 1).

### Morphological description of gregarines of *Schistocerca gregaria*

Young trophozoite stages (also referred to as cephalonts in historical publications [7, 17, 30]) (Fig. 1A) were observed in the two subspecies, regardless of the geographical location/raising facilities (Table 2). The globular epimerite with a short neck was visible in their anterior part (Fig. 1A). The density of infections could be very high, as shown by the number of trophozoites attached to the gut epithelium of an *S. g. gregaria* host from Morocco (Fig. 1B). The epimerite of attached trophozoites was enclosed in the host epithelial cell (Fig. 1C). High densities of trophozoites were also found in the ceca (data not shown) and midgut (solitary gamonts and syzygies (Fig. 1D)). The protomerite of trophozoites and gamonts was oval or slightly conical (Figs. 1A–1D); in syzygies, it appeared to be flattened at the top of the satellite with a ridge formed during pairing with the primite (Fig. 1D). Scanning electron microscopy revealed a similar ridge at the top of the satellite in *G. garnhami* syzygies [42]. The deutomerite was cylindrical or ovoid, and quite stocky in older trophozoites and syzygies (Fig. 1D). A constriction of the septum was visible between the posterior part of the protomerite and the anterior part of the deutomerite (Fig. 1D). The nucleus was seen in the opaque endocyte of the deutomerite. Longitudinal epicytic folds were visible at the surface of these trophozoite/gamont stages (Figs. 1A–1C). Thickening of the ectocyte was visible above the endocyte at the apex of the primite protomerite (Fig. 1D).

The gamonts in *S. g. flaviventris* from South Africa (*L* (length) = 402 ± 79 μm, *W* (width) = 172 ± 42 μm, *n* = 27) were very similar in size to gamonts in *S. g. gregaria* from Belgium (*L* = 410 ± 53 μm, *W* = 200 ± 39 µm, *n* = 34), but slightly smaller in *S. g. gregaria* from Morocco (*L* = 332 ± 43 μm, *W* = 96 ± 16 μm, *n* = 4). Moreover, smaller and much thinner trophozoites were observed in the latter (*L* = 192 ± 15 μm, *W* = 34 ± 4 μm, *n* = 12) (Fig. 1A). Also interestingly, gamonts in *S. g. gregaria* from Belgium were much stockier (*L*/*W* = 2.1 ± 0.2) than gamonts in *S. g. flaviventris* from South Africa (*L*/*W* = 2.4 ± 0.3) and gamonts (*L*/*W* = 3.5 ± 0.2) and trophozoites (*L*/*W* = 5.8 ± 1.0) in *S. g. gregaria* from Morocco. The ratios of protomerite (P) to deutomerite (D) lengths were, however, similar for gamonts, regardless of the infected hosts (P/D = 0.25 ± 0.04 (South Africa, *n* = 27); P/D = 0.23 ± 0.06 (Belgium, *n* = 34); P/D = 0.23 ± 0.07 (Morocco, *n* = 4), and also for the thinner trophozoites found in Moroccan *S. g. gregaria* specimens (P/D = 0.26 ± 0.04, *n* = 12). Overall, for trophozoites and gamonts infecting these hosts, regardless of the subspecies and their geographical location, the values were: *L* = 370 ± 98 μm; *W* = 159 ± 69; *L*/*W* = 2.83 ± 1.38 (*n* = 77).

Gametocysts in dehiscence were observed, producing ~8 (but sometimes more) pale orange basal discs, circular cellular structures with a central opening that eventually developed across the mucilaginous layer (ectocyst) into sporoducts with swollen bases (Fig. 1E). Their length was ~1/3 that of the diameter of the gametocyst (Fig. 1E). Gametocysts diameters were 350 ± 56 μm, *n* = 36 (from 210 to 420 μm). Oocysts extruding as chains through these sporoducts (Fig. 1F) were barrel-shaped with a thick wall enclosing eight sporozoites (Fig. 1G). Their size was quite uniform (*L* = 6.54 ± 0.32 µm, *W* = 4.32 ± 0.23 µm, *n* = 89) (Fig. 1G).

### Morphological description of gregarines of *Locusta migratoria*

Trophozoite stages attached to the gut epithelium of hosts were not seen, but a scar remained visible where the epimerite had been present at the top of the protomerite of detached gamonts (Figs. 1H–1I). These gamonts were rather cylindrical with a sub-globular protomerite, flattened and slightly constricted at the proto-deutomerite septum (Figs. 1H, 1J, 1K). The deutomerite was much longer and more slender towards the posterior end (Fig. 1H). The size of the gamonts varied but the mean size (*L* = 219 ± 48 μm, *W* = 93 ± 30 μm, *n* = 37) was smaller than the mean size observed in *S. gregaria* specimens (see above). Gamonts were also quite stocky (*L*/*W* = 2.5 ± 0.6, *n* = 37). In caudo-frontal syzygies, the protomerite was sub-globular in the primite, but shorter and flattened with a circular anterior edge in the satellite (Figs. 1J–1K). The deutomerite was cylindrical, slightly wider in the anterior part (Fig. 1J), ovoid in syzygies (Fig. 1K), with a rounded posterior end. The spherical nucleus could be seen in the opaque cytoplasm (endocyte) of the deutomerite (Fig. 1K). Longitudinal epicytic folds were seen at the surface of these stages (Figs. 1H–1J). The length of these syzygies was (*L* = 456 ± 73 μm, *W* = 93 ± 30 μm, *n* = 16) in our studies. The ratio of protomerite (P) deuteromerite (D) lengths was ~1/4 (P/D = 0.25 ± 0.05, *n* = 21). Gametocysts were spherical with a mucilaginous layer (ectocyst). Under this layer, and as observed in gregarines that infect *S. gregaria*, basal discs of the future sporoducts differentiated at the surface of encysted gametocysts. These basal discs were also orange with a central white aperture, but were fewer in number (< 8, *n* = 15). Like in the case of gregarines that infect *S. gregaria*, chains of oocysts were extruded through sporoducts (Figs. 1L–1M) whose length in gregarines of *L. migratoria* is longer and represents ~1/2 the diameter of the gametocyst (Fig. 1L). Gametocysts diameters were 227 ± 35 μm, *n* = 18 (from 190 to 296 μm). Oocysts, that were also emitted as chains from sporoducts, were also barrel-shaped with a double wall but were slightly longer and slimmer (*L* = 6.83 ± 0.27 μm, *W* = 3.99 ± 0.19 μm, *n* = 40, Fig. 1M) than the oocysts emitted by gregarines that infect *S. gregaria* (Figs. 1G, 1F).

### Statistical comparison of morphological measurements

For the gamonts, the means of the lengths (*p*-value = 2.2e-16; *df* (degree of freedom) = 111.97) and of the widths (*p*-value = 8.574e-11; *df* = 111.13) were significantly different between the gregarines infecting *S. gregaria* and *L. migratoria*. However, there were no significant differences between the length/width ratios between these two groups. Concerning the gametocysts diameters, the mean was significantly different (*p*-value = 1.986e-13; *df* = 49.386). Finally, for the oocysts, both mean length (*p*-value = 6.664e-07; *df* = 89.407) and mean width (*p*-value = 5.722e-13; *df* = 88.967) were significantly different.

### SSU rDNA sequences

**Figure 2:**
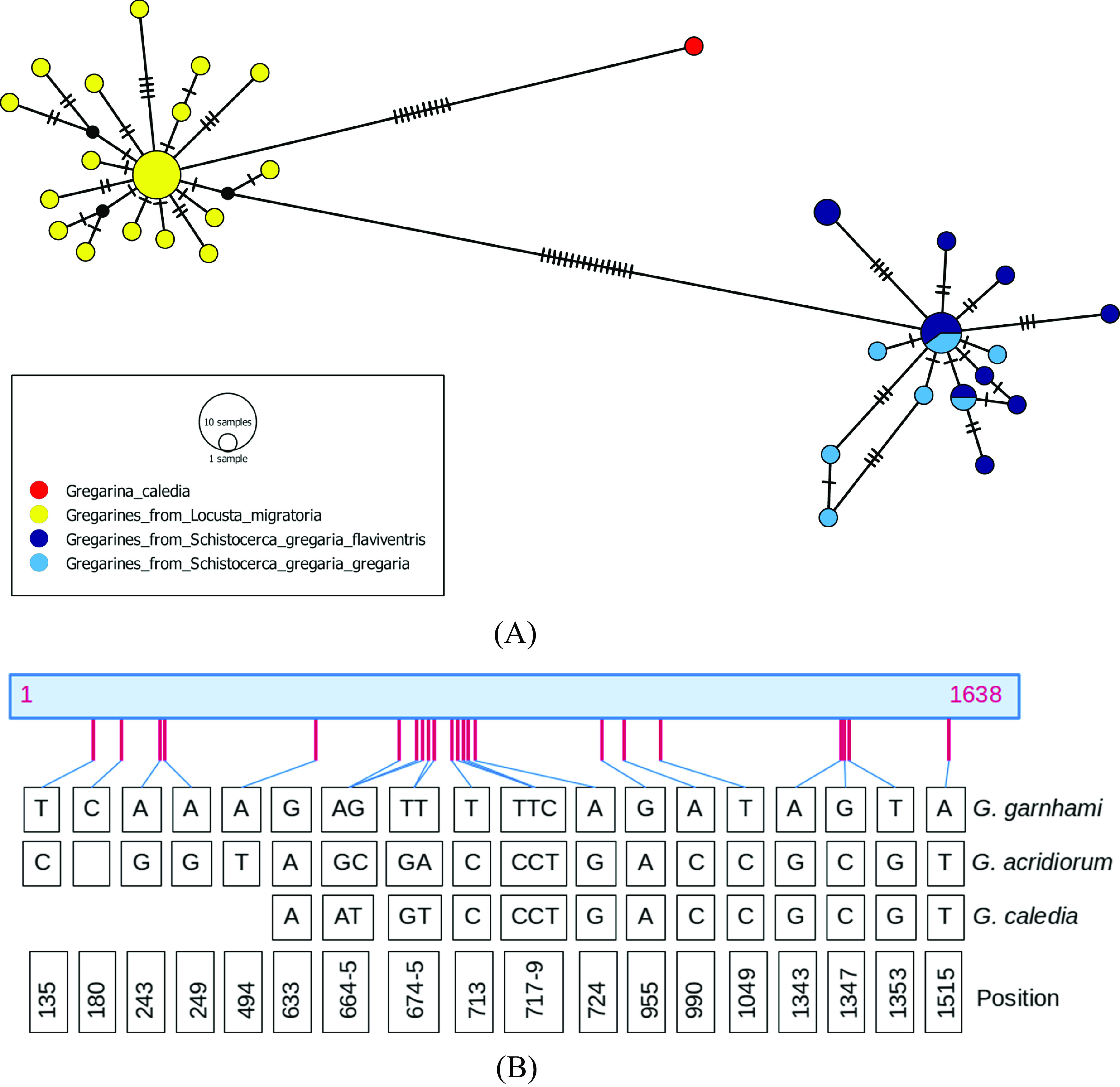
A: Minimum spanning network for the 43 cloned sequences of the SSU rDNA region studied, and the published sequence of *G. caledia* (L31799). Each link between haplotypes indicates one mutation, including indel events. The colors indicate the species or subspecies of the host. This network was inferred using POPART [25]. B: Distribution of the 22 polymorphic positions in SSU rDNA locus regions V1-V8 (1638-bp), between type 1 (presumably *G. garnhami*) (*n* = 20) and type 2 (presumably *G. acridiorum*) (*n* = 23) sequences, amplified from gregarines parasitizing respectively *S. gregaria* and *L. migratoria*. The corresponding positions are also given for *G. caledia* (L31799, 1210 bp) parasitizing *Caledia captiva*. Eleven additional positions, otherwise conserved between *G. garnhami* and *G. acridiorum* sequences, are modified in *G. caledia* sequence: site 1059, G deletion; sites 1161-1164: GAGC substituted by AG-G; site 1181: G substituted for C; site 1187: G substituted for A; sites 1231 and 1240: T substituted for C; site 1493: T insertion; site 1584: G substituted for A.

To further characterize these gregarines, a molecular study was designed to sequence most of the SSU rDNA locus from gamonts and gametocysts, isolated from several host specimens belonging to *L. migratoria* and two subspecies of *S. gregaria* (Table 2). A total of 23 sequences were generated from gregarines found in 7 specimens of *L. migratoria* on three collection dates, and 20 sequences were generated from gregarines found in five specimens of *S. gregaria* from a total of three geographical origins and/or raising facilities (Table 3). Regardless of the subspecies and the geographical location of hosts and their maintenance facilities, all the gregarines isolated from *S. gregaria* specimens shared the same “type 1” sequence (1638-bp long), presumably corresponding to *G. garnhami*, whereas all the gregarines isolated from *L. migratoria* specimens presented a clearly distinct “type 2” sequence (1637-bp long), presumably corresponding to *G. acridiorum*. Multiple sequence alignment and distance analyses were performed to qualify intra-species and inter-species variations, and clearly revealed two distinct clusters (Fig. 2A). Within the sequence group of gregarines from the host *S. gregaria*, the mean level of divergence was 0.2%, whereas within the sequence group of gregarines from the host *L. migratoria*, the mean level of divergence was 0.3%. The mean level of genetic distance between gregarine sequences from *S. gregaria* and those from *L. migratoria* was 1.5%, whereas the genetic divergence from *G. caledia,* parasite of *C. captiva*, was 1.1% with the gregarine group from *L. migratoria*, but 2.2% with the gregarine group from *S. gregaria*. In all, 22 conserved polymorphic positions, rather evenly distributed along the SSU rDNA locus, were identified between “type 1” and “type 2” sequences (assumed to be *G. garnhami* and *G. acridiorum*, respectively), as schematized in Figure 2B.

### Phylogenetic analysis

**Figure 3:**
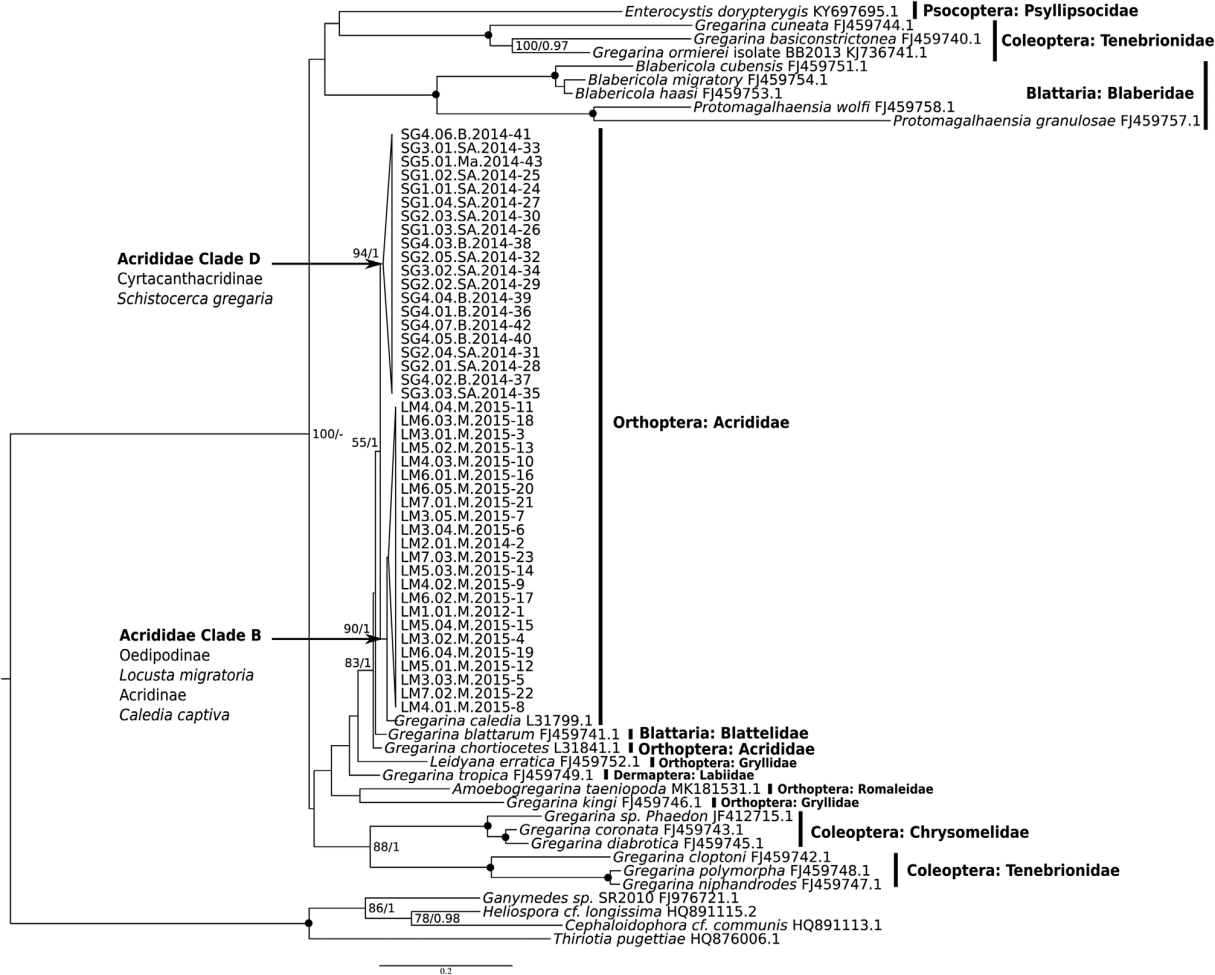
Phylogenetic tree built using 69 SSU rDNA sequences for 1,433 sites in order to zoom in on the clade Gregarinoidea including gregarines parasites of Orthoptera [11]. Outgroup consists of 4 sequences from Cephaloidophoroidea species that infect crustaceans, currently considered as the sister group of Gregarinoidea [29]. Evolutionary history is inferred by maximum likelihood and Bayesian inference using a GTR substitution model with gamma-distributed rate variation across sites plus invariant sites. Maximum likelihood topology is shown, with supports from both methods. Bootstrap < 75% and posterior probabilities < 0.95 are not shown. Black spots indicate 100/1 supports. The gregarines infecting *L. migratoria* clustered with *G. caledia*, isolated from the grasshopper *Caledia captiva* [30], the gregarines infecting *S. gregaria* forming a distinct independent clade. *G. chortiocetes,* infecting the locust *Chortiocetes terminifera* [30], and *Gregarina blattarum*, infecting the cockroach *Blatella germanica* [11] form sister branches to this group. The taxonomy of locust hosts is indicated, as established by Song et al, [38].

A phylogenetic approach, using partial SSU rDNA sequences and both maximum likelihood and Bayesian inference reconstructions, indicated that gregarine sequences from the two different host species studied clustered with sequences from other Gregarinoidea species (as described in [9, 11, 35]) with a high ML bootstrap value and Bayesian posterior probability (Fig. 3). These novel gregarine sequences form two clearly distinct clades according to their host species, and it thus appears that all *S. gregaria* hosts, regardless of their subspecies and the geographical location at which they were maintained, were infected by the same species (based on their SSU rDNA sequence) that was clearly distinct from the parasitic species infecting *L. migratoria.* The SSU rDNA sequence from *G. caledia* showed closer affinity to gregarine sequences from the host *L. migratoria* than from the host *S. gregaria* (see also Fig. 2). Furthermore, we observed that hosts of the “type 2” (presumably *G. acridiorum*) and *G. caledia* sequences, i.e. *L. migratoria* and *C. captiva*, belong to the same clade B of the acridian phylogeny as defined by Song et al. 2018 [38], while *S. gregaria*, infested with *G. garnhami* (“type 1” sequences), belongs to a distinct clade D, as indicated in Figure 3. Thus, gregarine distribution appears to parallel the taxonomy of these three hosts. This observation will however need to be confirmed, as the ML bootstrap support remains low (55), despite high Bayesian posterior probability (Fig. 3).

## Discussion

To determine whether the acridian orthopterans *S. gregaria* and *L. migratoria* are infected by the same gregarine species, their parasites were isolated and morphological and molecular analyses were performed using a series of host specimens of both species collected from a range of different locations and insect raising facilities (Table 2). While morphological investigations confirmed highly similar parasites with only tenuous morphological and behavioral differences, molecular investigations yielded unambiguous results strongly supporting different gregarine species in these *S. gregaria* and *L. migratoria* hosts.

### Molecular data support distinct species

Molecular characterization, based on the partial SSU rDNA marker (V1–V8 region [18]) of all gregarines studied, unambiguously demonstrated that all *S. gregaria* hosts – regardless of their subspecies and raising facilities – are infected by the same gregarine species (presumably *G. garnhami*), whereas all *L. migratoria* hosts are infected by a distinct species (presumably *G. acridiorum*). Both gregarine sequences clustered in the previously reported Gregarinoidea clade [11, 29, 31]. Overall, 22 different bases were identified in this 1638 bp region that could be used to delimit the species. The 1.5% genetic distance between the two sequences is in agreement with previously described inter-specific levels of genetic divergence that, for example, distinguish *Gregarina niphandrodes* from *Gregarina polymorpha* (1.44%) [31]. However, it should be noted that, according to the same authors, such “low” genetic divergence could also correspond to “intra-specific” variability [31]. Certainly, additional studies will be needed to clarify this issue, but we recently demonstrated that two marine gregarines with an almost identical SSU rDNA sequence (1 bp difference for 1702 positions, i.e. ~0.05% divergence) displayed ~10% overall nucleic acid divergence at the genomic level, preventing genetic crossing, i.e. arguing for different species (I. Florent and J. Boisard, unpublished data).

Based on these molecular results and on data in the literature, notably the identification of their hosts, we propose that the “type 1” sequence found in gregarines infecting *S. gregaria* hosts may correspond to the species named *G. garnhami*, reported by several authors and collected from *S. gregaria* [7, 42]. The gregarine species found in *L. migratoria* likely corresponds to *G. acridiorum*, in agreement with Léger [24], but not with the proposal of Lipa et al. [27].

### Some morphological and behavioral features discriminate the two species

To further confirm that two distinct gregarine species infect *S. gregaria* vs. *L. migratoria*, we focused on their possibly discriminating morphological and behavioral differences. Several morphological characters have been proposed in the literature to discriminate acridian gregarines, including: (1) the number and length of sporoducts, (2) the size of oocysts, and (3) the presence of a sharply pointed posterior extremity in *G. garnhami* versus a rounded extremity in *G. acridiorum* gamonts (see Table 1), even though, as indicated by Lange and Wittenstein, 2002, “such morphological features are probably not sufficient to delimit species, as very similar values in ranges and ratios were found between them” [22].

The sporoducts were indeed shorter in gregarines that infect *S. gregaria* (Fig. 1E) than in gregarines that infect *L. migratoria*, (Fig. 1L), supporting the hypothesis that *S. gregaria* can be infected by *G. garnhami* (~1/3 of the diameter of the gametocysts, Table 1) and *L. migratoria* by *G. acridiorum* (~1/2 of the diameter of the gametocysts, Table 1). The comparative study of sizes of barrel-shaped oocysts led to a less definitive indication. In gregarines that infect *S. gregaria*, the measurements (6.54 ± 0.32 μm × 4.32 ± 0.23 μm, *n* = 89) closely matched those reported in the literature for *G. garnhami* (6.5–7 μm × 4 μm, Table 1), compared to the remaining four species (Table 1). In gregarines that infect *L. migratoria*, these measurements (6.83 ± 0.27 μm × 3.99 ± 0.19 μm, *n* = 40) somewhat resemble those proposed in the literature for *G. acridiorum* (7.6 × 3.3 μm, Table 1), but are also very similar to the values reported for *G. garnhami* (6.5–7 μm × 4 μm, Table 1). However, these measurements are clearly more distantly related to the measurements reported for oocysts of the three other morphologically similar species: *G. rigida* (8 μm × 5 μm), *G. ronderosi* (5 μm × 3.2 μm), and *G. caledia* (12 μm × 6 μm) (Table 1).

However, the sharp (*G. garnhami*) versus round (*G. acridiorum*) posterior extremity of gamonts, proposed as a distinguishing feature between these two species, was not always reliably observed in our study and was therefore not retained as a distinguishing feature. Also, the number of sporoducts per gametocyst, currently reported in the literature to be larger in *G. acridiorum* (12–15) than in *G. garnhami* (8) (see Table 1), does not support our hypothesis that *G. acridiorum* is present in *L. migratoria* and *G. garnhami* is present in *S. gregaria*, as we observed the contrary: the number of sporoducts was less than eight for gregarines infecting *L. migratoria* (Fig. 1L) and more than eight for gregarines infecting *S. gregaria* (Fig. 1E). However, as previously mentioned by Clopton et al., 2009, the number of sporoducts is probably an unreliable taxonomical character [11]. Gametocysts diameters were also larger in *G. garnhami* (350 ± 56 μm, *n* = 36) vs. *G. acridiorum* (227 ± 35 μm, *n* = 18), but with overlapping values (210–420 μm for *G. garnhami*; 190–296 μm for *G. acridiorum*).

In the course of this study, we identified a third distinctive feature that is rarely mentioned in the literature: the fact that gregarines were systematically observed in the ceca of *S. gregaria* but never in the ceca of *L. migratoria*. The presence of *G. garnhami* but also *G. rigida*, *G. ronderosi* and *G. caledia* in the ceca of their hosts has also been systematically reported (Table 1) but interestingly, only the midgut was reported to be infected in the host specimens examined by Léger 1893, which included *L. migratoria* [24]. Whether this behavioral difference results from differences between gregarine species, in terms of ecological niche or host-parasite relationship, or from anatomical specificities in the two infected hosts, as already suggested [4], needs to be investigated experimentally. This third difference further supports the hypothesis that the two gregarines that infect either *S. gregaria* or *L. migratoria* should be considered distinct species. Remarkably, the gregarines recorded by Lipa et al. [27] in different acridian species, developed in the midgut but also in the gastric intestinal ceca of their hosts, a habitat that could indicate that they were infected by *G. garnhami* rather than by *G. acridiorum*. Alternatively, these acridian species may have hosted entirely novel (cryptic) gregarine species that remain to be characterized.

In addition to the morphological and developmental differences described above, these two gregarines share many peculiarities such as the ectocyst and the orange basal discs involved in gametocyst encystment then dehiscence [17]. The ectocyst, which designates the thick outer gelatinous layer or translucent hyaline coat of the gametocyst, is found in a wide range of gregarines of Orthoptera and is probably an adaptation to the host environment that makes it possible to keep the developing gregarine in a moist atmosphere [17]. Basal discs, involved in the extrusion of the sporoducts of all gregarines belonging to the superfamily Gregarinoidea, are widely observed in Hexapoda hosts [17]. The basal discs are orange in all the gregarines of Orthoptera and the gamonts are often pale yellow, as we observed here for gregarines infecting both *S. gregaria* and *L. migratoria* hosts. Importantly, it is possible that these morphological features are the product of plasticity, so their taxonomical significance remains to be explored.

### Taxonomic consequences

Based on these differences and on the available literature, we thus endorse the hypothesis that the species that infect *S. gregaria* should bear the species name *G. garnhami,* in agreement with the morphological characters established for this species (Table 1) and in agreement with a previous proposal by Valigurova and Koudela [42]. Indeed, these authors already disputed the interpretation of Lipa et al. (1996) [27] arguing that in their studies, Lipa et al. did not observe the developmental stages that are able to differentiate these species, i.e. the number and length of the sporoducts involved in the dehiscence process and the size and shape of their oocysts [42]. Concerning the species that infect *L. migratoria,* we maintain our proposal to name them *G. acridiorum*, even though only in partial agreement with the morphological characters established for this species (Table 1). This proposal is logical given the taxonomic history of this species, as the first *Gregarina* species found to infect *L. migratoria* was called *Gregarina (Clepsidrina) acridiorum* [21, 24], and the absence of colonization of the hosts’s ceca. Although the size and shape of the oocysts we observed in the gregarine infecting *L. migratoria* do not perfectly match the measurements reported for *G. acridiorum* in the literature (Table 1), the size and shape of the oocysts we observed in gregarines that infect *S. gregaria* perfectly match the measurements reported for *G. garnhami* in the literature. However, the oocyst in gregarines found in *L. migratoria* were clearly thinner and longer than the oocysts in gregarines found in *S. gregaria*, observed in similar conditions. The observed length of sporoducts also agrees with data reported for both species in the literature, unlike the observed number of basal discs/sporoducts developing at the surface of gametocysts in dehiscence (Table 1). As mentioned above, this point should be interpreted with caution as it has been reported that the number of basal discs and the development of sporoducts may vary according to environmental conditions (temperature, hygrometry) as well as possibly the size of the gametocysts [7, 11].

### Morphological plasticity and host conditions

The morphological data showed that the developmental stages of the gregarines infecting *S. gregaria* (Figs. 1A–1G) were generally very similar, though slightly longer and larger than the developmental stages of the gregarines infecting *L. migratoria* (Figs. 1H–1M). However, depending on the raising facility and/or geographic origin, gregarines – notably trophozoites and gamonts – appeared to be slimmer in *S. g. gregaria* hosts from Morocco and *S. g. flaviventris* hosts from South Africa (not shown) than in gregarines infecting *S. g. gregaria* hosts from Belgium (Fig. 1D). The latter, which were much stockier, were more similar to the gamont stages of the gregarines that infect *L. migratoria* (Figs. 1H–1K). Since *S. g. flaviventris* hosts and *S. g. gregaria* hosts from the South African and Moroccan facilities, respectively, were also observed to be unhealthy (mature adults behaved sluggishly and seemed soft and light from food), while *S. g. gregaria* hosts from the Belgium facility and the *L. migratoria* hosts maintained in France did not seem to be particularly affected by the presence of their infecting gregarines (see also Table 2), we favor the hypothesis that environmental differences or co-occurring microorganisms may explain the difference in “fitness” between “African” and “European” hosts, as this was not due to infections by distinct gregarine species.

### How many distinct species are there for these gregarines?

The gregarine developmental stages described in *S. gregaria* and *L. migratoria* hosts are very similar morphologically, and share many characteristics including the thick mucilaginous ectocyst of the gametocyst, orange basal discs associated with great variability of size parameters. As these morphological features have also been observed in other species, particularly in *G. rigida*, *G. ronderosi* and *G. caledia* collected from different (and sometimes from identical) orthopteran hosts (Table 1), these species need to be further characterized at the molecular level to solve their phylogenetic relationships. The only molecular sequence available (*G. caledia*, L31799) although rather small (1210 bp) strongly suggests a third distinct species, closely related phylogenetically to the proposed *G. acridiorum* but still with some observed genetic distance (1.1%). *Gregarina caledia* is also potentially morphologically distinguishable by the larger size of its oocysts and its ability to infect host ceca (Table 1, [30]). Importantly though, in the first morphological reports, this species was said to be closely related to *G. garnhami* with which it also shares the ability to infect host ceca [30].

*Gregarina rigida* (Hall, 1907) Ellis, 1913, has also been reported in a range of orthopterans. When describing this species, the authors did not cite any literature on *G. acridiorum*, so, in 1968, Corbel concluded that *G. rigida* was a junior synonym of *G. acridiorum* [14]. To be confirmed, the status of this species (e.g. synonym of *G. acridiorum*?) therefore requires molecular data, even though available measurements of oocysts and the fact that it has also been found in host ceca (Table 1) favor a distinct species. Importantly, in 2002, *G. ronderosi*, which was found in the argentine grasshopper, *Dichroplus elongatus*, was named a novel species by Lange and Wittenstein due to the lack of infection in specimens of *L. migratoria* experimentally infected with this gregarine [22]. It thus also possibly corresponds to a fifth distinct species, also awaiting molecular characterization. Lange and Wittenstein, 2002, even suggested that *G. ronderosi* could be synonymous with *G. garnhami*, but that molecular data were required as morphometric differences did not enable conclusive delimitation of the species [22].

## Conclusion

It is well documented that assigning protist species can no longer rely on morphological information alone, but should include molecular data in an integrated taxonomic approach [5, 6]. The data presented here confirm that most morphological and morphometric differences cannot conclusively delimit closely related species, while molecular data can reveal clearly measurable differences. By strongly suggesting that *S. gregaria* is infected by *G. garnhami*, whereas *L. migratoria* is infected with *G. acridiorum*, our data suggest two important discriminating features: the respective size of the oocysts of *G. garnhami* and *G. acridiorum*, but also their location in their respective host’s gut. The first consequence is that *G. garnhami* can no longer be considered a junior synonym of *G. acridiorum,* contrary to the proposal by Lipa et al. [27] and is therefore reinstated here as a valid taxon, in agreement with the proposal of Valigurova and Koudela [42].

The exact distribution of *G. garnhami* and *G. acridiorum* in Orthoptera remains to be further investigated at this stage as clearly, when synonymized, they were assumed to infect the same series of host species [17]. Additional studies, specifically molecular studies, are crucial to determine the diversity of gregarine species that infect acridians, beyond the establishment of morphological specificities (see Table 1). This could help determine whether *G. rigida* and *G. ronderosi* are in fact distinct species or should be synonymized with other species. Interestingly, *G. caledia*, a parasite of the Australian locust *C. captiva* reported to be very similar to *G. garnhami* and for which molecular data are available [30], should be considered a species distinct from both *G. garnhami* and *G. acridiorum* as argued in this paper. Based on our molecular studies, *G. caledia* presents closer phylogenetic similarity to *G. acridiorum* (Fig. 3). A major challenge concerns the precise diversity of the species *G. acridiorum* that has been described in over 60 orthopteran hosts, from both the Caelifera and Ensifera orders, as is also the case for *G. rigida*. It is likely that these two species correspond to a much greater diversity of probably cryptic species that remain to be described by this type of integrative taxonomical approach, in the diversity of their currently described hosts.

## Conflict of interests

The authors declare that they have no conflict of interest.
